# Supplementary material for: First-line infertility treatment in normal or subnormal sperm: Interest of a simplified pre-IMSI test
Source: PLoS One. 2024 Jul 15;19(7):e0307080. doi: 10.1371/journal.pone.0307080 (PMC11249273; doi:10.1371/journal.pone.0307080)
Supplement: Attachment — Submitted filename: Reponse to reviewers .docx [file pone.0307080.s002.docx]

**Journal Requirements:**

1. Please ensure that your manuscript meets PLOS ONE's style requirements, including those for file naming.

Thank you for this remark.

Modifications have been done.

**Reviewer #1:**

1. the number of infertile couples included in the study and thereafter in the different groups of ART and secondly of PIT score. This parameter is insufficiently discussed and taken into account. The selected population for PIT analysis is very heterogeneous form repeated ART failures, repeated miscarriages, abnormal embryonic development.

Thank you for your comment.

Details of the small number of couples included in the different groups and subgroups are explained and discussed in the following lines:

- Lines 453-455 : *« […] although these differences between the three PIT subgroups were statistically not significant potentially because of a lack of statistical power.”*
- Limitations paragraph, lines 494-502 :*« Our study has certain limitations. Firstly, the heterogeneity of the numbers studied within the different ART techniques and PIT groups means that some analyses were carried out on small sample sizes, which do not always guarantee sufficient statistical power to highlight a difference of interest. Our results should therefore be taken with caution and confirmed by larger studies. Similarly, ART techniques were only compared within the PIT groups on the criterion of clinical pregnancy, and not on the criterion of live birth, which is the real aim for infertile couples. In our study, the number of live births was insufficient to allow analysis, and it is difficult to state with certainty that what is observed for clinical pregnancies will also be observed for live births. Here again, our results need to be confirmed by larger studies.”*
- Lines 516-517 : *Conclusion : « The number of transfers in each ART and PIT score groups remains low and obliges us to remain cautious in interpreting these preliminary results.”*

2. The methodology for MSOME analysis is insufficiently described. The authors did not describe how they differentiate large vacuoles from small ones. These different parameters should be addressed.

Thank you for your remark.

- An explanation has been added in lines 202-203 : *« Evaluation of vacuole size (small, intermediate, large) was semi-quantitative, as described in Fig 1. “*

- Fig 1 has been modified for greater clarity (see Fig 1) in lines 205-208: *«* ***Fig 1. Pre-IMSI test (PIT): grades of spermatozoa according to the number and size of vacuoles.*** *Grade A sperm cells either presented a maximum of two small vacuoles (a) or an intermediate vacuole (b). Grade B sperm cells had either more than two small vacuoles or at least one large vacuole (c).”*

3. It should be noticed that it is difficult for the reader to find what is a subnormal semen sample. This parameter should be addressed

We agree with your comment and have now added an explanation in lines 156-158 : *« Subnormal sperm may present one or more minor abnormalities of the spermogram-spermocytogram, but at least 1 million progressive spermatozoa were available after selection”.*

4. The analysis of semen parameters according to WHO should be more precisely described

Thank you for this comment.

The subtitle « Semen samples and quality assessment » has been completed lines 174-186.

5. Considering ART, IVF and ICSI cycles, no information is available concerning the ovarian stimulation, number of FSH units, estradiol at trigger, number of top quality embryos, the classification used for embryo grading for IVF, ICSI and IMSI

Thank you for this comment.

The parameters reported in this article were those that might have a direct influence on the results (patient's age and ovarian reserve status (AMH)) and are the most strongly correlated with IVF birth rates. We based this analysis on the following points :

- Ovarian stimulation:
  - the efficacy of the agonist and antagonist protocols is comparable in hyper-responders, normo-responders and poor-responders according to ESHRE (Ref A below). Intrauterine inseminations were carried out on stimulated cycles.
- According to the litterature :
  - the number of FSH units is not correlated with the attempt outcome in poor responders (Ref B below).
- Estradiol level at induction may be correlated to the risk of hyperstimulation, but not to the birth rate after fresh embryo transfer.

According to the literature, the parameters you asked for are not systematically included, as in the paper by Asali *et al.* (Ref C, below), which is similar to ours.

Embryo classifications have been added to the materials and methods, lines 241-245 : « *Cleaved embryo quality was estimated 44 to 46 hours post-fertilization. The embryo score (Blefco classification (44)) was established on Day 2 (44 ± 1 hours post- fertilization) or at the 8-cell stage on Day 3 (68 ± 1 hours post- fertilization). Blastocysts were evaluated on Days 5 and 6 according to the Gardner classification (45).”*

***Ref. A :*** *Ovarian Stimulation TEGGO, Bosch E, Broer S, Griesinger G, Grynberg M, Humaidan P, Kolibianakis E, Kunicki M, La Marca A, Lainas G, Le Clef N, Massin N, Mastenbroek S, Polyzos N, Sunkara SK, Timeva T, Töyli M, Urbancsek J, Vermeulen N, Broekmans F. ESHRE guideline: ovarian stimulation for IVF/ICSI†. Hum Reprod Open. 2020 May 1;2020(2) ; Erratum in: Hum Reprod Open. 2020 Dec 29 ;2020(4)*

***Ref. B :*** *Liu X, Wen W, Wang T, Tian L, Li N, Sun T, Wang T, Zhou H, Zhang N, Qu P, Mol BW, Li W, Shi J. Increased versus standard gonadotrophin dosing in predicted poor responders of IVF: an open-label randomized controlled trial. Hum Reprod. 2022 Jul 30;37(8):1806-1815.*

***Ref. C :*** *Asali A, Miller N, Pasternak Y, Freger V, Belenky M, Berkovitz A. The possibility of integrating motile sperm organelle morphology examination (MSOME) with intracytoplasmic morphologically-selected sperm injection (IMSI) when treating couples with unexplained infertility. PLoS One. 2020; 15(5)*

6. The females confounding factors during ovarian stimulation are not taken into account

Thank you for this comment.

We studied patient age, AMH, BMI, infertility period and etiology of infertility, which are the main female confounding factors. As shown in Table 1, all the main female characteristics were identical, except for the PIT score II and III subgroups in which we had more patients with PCOS that might explain some of the miscarriages.

If we refer to ref. D. below, the "total number of embryos, number of injected oocytes, cause of infertility, female age, and polycystic ovary syndrome (PCOS) were the most important factors predicting successful delivery."

***Ref. D :*** *Amini P, Ramezanali F, Parchehbaf-Kashani M, Maroufizadeh S, Omani-Samani R, Ghaheri A. Factors Associated with In Vitro Fertilization Live Birth Outcome: A Comparison of Different Classification Methods. Int J Fertil Steril. 2021 Apr;15(2):128-134.*

7. When considering the main clinical outcomes, the authors reported the global percentage rate at their center. However, they did not describe their reference population (sample size, females and males characteristics, number of ART cycles, ...)

Thank you for this comment.

Comparing the study results with those of our Center during the same period allows for better interpretation. We do not have defined a reference population. We always compare ourselves to national or international results published by the French Biomedecine Agency or in the literature, respectively. Our center results are in line with national standards.

However, we added in Table 3 the number of cycles/transfers, miscarriages and clinical pregnancy rates in our Center during the same period as the study. We also added the following sentence lines 331-334 : *« In order to better interpret our study results, the last column of Table 3 shows the global percentage of all attempts at our ART Center during the study period (Feb 2017 - Oct 2020). The only exclusion criterion of this reference population was women over 39 years of age.”*

8. In Table 3, they also presented the number of transfers but they did not present for IVF, ICSI and IMSI the number of cycles of ovarian puncture. Consequently, the presentation of the data of the ART cycles did not respect the conventional presentation generally proposed in most of papers.

Thank you for this comment.

We agree with your point of view and have added the number of cycles in Table 3.

We have deliberately expressed pregnancy/birth/miscarriage rates per transfer rather than per cycle, as expressing results per cycle makes it difficult to conclude as a failure before all embryos have been transferred.

9. The reviewer was very surprised by the miscarriage rate who was very higher in the different Pit score and IUI, ICSI and IVF

Thank you for this comment.

The miscarriage rates were a real surprise for us too when we analyzed the results.

That's why we looked at our global results during the same period and reported them in Table 3, to show that these high miscarriage rates were not a bias in the study, but a particularity linked to the patients included in this study.

10. The conclusion of the advantage of one technique from another based on the PIT score should be discussed carefully because the number of transfer in each ART and PIT score groups remains very low.

Thank you for this comment.

The discussion and conclusion have been modified in this sense, and a paragraph on the study limitations has been added.

11. Major revisions are required mainly in the materials and methods and results parts. The discussion part should have a part on strength and limitation of the study

We agree entirely with your comment and have now added paragraph on the.study limitations in lines 494-502 : « *Our study has certain limitations. Firstly, the heterogeneity of the numbers studied within the different ART techniques and PIT groups means that some analyses were carried out on small sample sizes, which do not always guarantee sufficient statistical power to highlight a difference of interest. Our results should therefore be taken with caution and confirmed by larger studies. Similarly, ART techniques were only compared within the PIT groups on the criterion of clinical pregnancy, and not on the criterion of live birth, which is the real aim for infertile couples. In our study, the number of live births was insufficient to allow analysis, and it is difficult to state with certainty that what is observed for clinical pregnancies will also be observed for live births. Here again, our results need to be confirmed by larger studies.”*

12. The conclusion should be more nuanced

The conclusion has been modified accordingly.

**Reviewer #2:**

1. Could the authors please include some further representative images in Figure 1 for Grade A and B? Specifically could you show the range of severity for Grade B? Also it would be useful to highlight some cells that would fit the category ‘teratozoospermic but with normal PIT score’, this was slightly unclear. It would be more useful to have this figure ahead of the first results table in the results text or in the methods, rather than later in the discussion since you already discuss the grades in the methods section.

Thank you for this remark.

Fig 1 has been modified for greater clarity (see Fig 1) in lines 205-208: *«* ***Fig 1. Pre-IMSI test (PIT): grades of spermatozoa according to the number and size of vacuoles.*** *Grade A sperm cells either presented a maximum of two small vacuoles (a) or an intermediate vacuole (b). Grade B sperm cells had either more than two small vacuoles or at least one large vacuole (c).”*

2. The potential relationship between sperm epigenetic profiles and vacuoles will be new to many readers but the authors give this almost as an afterthought to the study in the last paragraph of the discussion. I think a further expansion of the literature around vacuoles and why they might help to predict success of assisted reproduction is warranted in the introduction. Especially given the controversy of sperm vacuoles in the field, it would be good to provide further explanation up front.

Thank you for this comment.

Modified lines 93-104 : *The origin of nuclear vacuoles in male infertility remains also widely debated (11). Early reports suggest that sperm head vacuoles may be due to DNA fragmentation (12–17), DNA decondensation (18–20) or exposure to reactive oxygen species (ROS) (21,22). Previous results (12,13,15–17,20,23,24) show that the DNA fragmentation rate may reflect the size and number of vacuoles,* *though the role of vacuoles in the process of DNA fragmentation is not yet fully understood. Moreover, spermatozoa with vacuoles have been shown to be associated with chromatin disorganization (abnormal chromatin condensation, aneuploidy, modification of spatial and chromosomal positioning) (2,13,18,25,26). A potential association between spermatozoa genomic stability and vacuolar morphology and location was also published (27) as well as the potential link between vacuolated spermatozoa and some epigenetic marks involved in chromatin condensation (28,29). Conversely, some studies argue that nuclear vacuoles result from a natural physiological process unrelated to DNA fragmentation (30,31).”*

3. Rating of the PIT scores – Just an observation but It was not intuitive to me to have PIT score I as poorest quality and PIT score III as highest quality. Perhaps something to consider ahead of publication.

We are agreed with your comment.

Modified lines 222-224 : *« At this stage, PIT score I was considered as the worst quality, PIT score II as of intermediate quality and PIT score III as the best.”*

4. In general, we face in the field that many interventions in the clinic may help to improve or predict pregnancy rate but do not necessarily translate to live birth outcomes. Although your study is still very meaningful with approaching significance, could you please comment on why this may be the case for your specific study and how your approach could be further improved? This can be included in the discussion.

You're right. It would has been interesting if we had also analyzed live births in Table 4. However, the numbers involved were insufficient.

Moreover, the literature describes a reduction in the number of miscarriages and malformations after IMSI. We thus preferred to focus on clinical pregnancies (Ref E and F below)

We are currently preparing a prospective study involving the use of our PIT score in the day-to-day life of our laboratory to complete the present results.

***Ref E :*** *Cassuto G, Hazout A, Bouret D, Balet R, Larue L, Benifla JL et al. Low birth defects by deselecting abnormal spermoatozoa before ICSI. Reprod Biomed Online. 2014 28:47-53.*

***Ref F :*** *Hershko-Klement A, Sukenik-Halevy R, Biron Shental T, Miller N, Berkovitz A. Intracytoplasmic morphologically selected sperm injection and congenital birth defects : a retrospective cohort study. Androl. 2016. 4:887-893.*

5. The authors state that a part of the purpose of the study is to increase efficiency/decrease time take for scoring. Perhaps it is useful then to indicate how long it takes to score a sample on average? If I was reading this and considering taking up the technique I would like to know how long it takes once well optimised.

Thank you for this comment.

Lines 402-403 has been modified accordingly : *« On average, it takes two observers 15 minutes to complete the test.”*

6. Most of the first paragraph and also second paragraph of the discussion is a repetition of the methods section, it should be revised.

Thank you for this comment.

Paragraphs 1 and 2 have been merged. Lines 388-426.

7. Could you please comment on the relationship between sperm vacuoles and miscarriage. Is this also potentially related to epigenetic status of the sperm?

Thank you for these question.

The following lines have now been modified:

- *98-103 : « Moreover, spermatozoa with vacuoles have been shown to be associated with chromatin disorganization (abnormal chromatin condensation, aneuploidy, modification of spatial and chromosomal positioning) (2,13,18,25,26). A potential association between spermatozoa genomic stability and vacuolar morphology and location was also published (27) as well as the potential link between vacuolated spermatozoa and some epigenetic marks involved in chromatin condensation (28,29).”*
- *455-459 : “All nuclear alterations of vacuolated spermatozoa described in the introduction can negatively influence male fertility and embryonic development, induce miscarriages (a decondensed DNA is more vulnerable), and eventually health problems in the offspring (29,49). Indeed, IMSI appears to significantly increase blastocyst development (5), decrease the incidence of structural defects (50) and miscarriage (51–56) even if the benefits of IMSI are still debated (57,58).”*

8. Line 424 – Reversely can be replaced with conversely

Word removed.

9. Line 451 vs line 434 – can you please double check the consistency of this recommendation. In line 434 you suggest PIT score III would recommend IVF or ICSI but then in line 451 you suggest IVF or IUI. I think based on the IUI results you present in the tables it would be difficult to recommend this but either way these statements need to be consistent for your recommendation to be meaningful.

We agree with your comment.

Harmonization between the two paragraphs. Part of the text has been moved to the conclusion - lines 519-522 : *« IMSI appears to be the technique of choice when sperm belongs to subgroup I, ICSI when it belongs to subgroup II and IVF when sperm is of subgroup III quality. If IUI is indicated, it is preferable to perform this technique mainly on subgroup III sperm.”*

10. It is good practise that you have included the data in Excel for publication. Please consider the utility of your column headings. It would help to provide a key in a second excel sheet/tab for certain terms that might need explanation.

Thank you for this remark.

A second excel sheet/tab has now been added.
